# Comparison of trapping methods for use in surveys for potential *Culicoides* vectors of orbiviruses

**DOI:** 10.1186/s13071-021-05059-9

**Published:** 2021-11-03

**Authors:** Michael Becker, Jeong-Seok Park, Glen Gentry, Claudia Husseneder, Lane Foil

**Affiliations:** 1 Agricultural Experiment Station, Department of Entomology, Louisiana State University Agricultural Center, 402 Life Sciences, Baton Rouge, LA 70803 USA; 2 1S1-5 203a, Chungbuk National University, 1 Chungdae-ro, Seowon-gu, Chengju, Chungbuk 28644 South Korea; 3 Agricultural Experiment Station, Bob R Jones Idlewild Research Station, Louisiana State University Agricultural Center, 4419 Idlewild Road, Clinton, LA 70722 USA

**Keywords:** Bluetongue virus, *Culicoides*, Epizootic hemorrhagic disease virus, White-tailed deer

## Abstract

**Background:**

Bluetongue virus (BTV) and epizootic hemorrhagic disease virus (EHDV) are orbiviruses that can cause fatal vector-borne diseases in white-tailed deer (*Odocoileus virginianus)*. Trapping methods for collecting potential *Culicoides* vectors of orbiviruses were compared to optimize surveillance studies.

**Methods:**

The number of captured midges and the virus infection rates of midge pools were compared for dry ice-baited Centers for Disease Control and Prevention (CDC) traps with or without black light. The number of individual midges of different *Culicoides* species captured at different crepuscular and nocturnal periods using rotator traps also was determined. The number of species/specimens of *Culicoides* was measured using five different trap methods including three animal-baited methods, a CDC trap with black light, and a CDC trap with no light.

**Results:**

In trial one, there was no significant difference (*P* = 0.37) in the proportion of BTV-infected flies caught in traps with light compared to traps without light. However, there was a significant difference (*P* = 0.026) for EHDV-infected flies, and 89% were captured in traps with light. In trial two, more specimens of *C. debilipalpis* were captured in the morning hours (06:00–08:00) than in the evening hours (18:00–20:00). For trial three, the animal-baited traps did not capture any species of *Culicoides* that were not captured in the CDC light traps. There was no significant difference (*P* = 0.22) in total specimens captured among all five trap types.

**Conclusions:**

Specimens of *Culicoides* infected with BTV were not repelled by light traps in the first trial, while the majority of the specimens positive for EHDV were caught in traps with light. For the second trial, specimens of *C. debilipalpis* were most abundant during early morning hours, and thus spray applications of insecticides for control of that species may be more effective at sunrise rather than sunset. For objective three, no animal-baited trapping method collected different species of midges when compared to the CDC traps with light, which is unlike certain studies conducted in other geographical regions.

**Graphical abstract:**

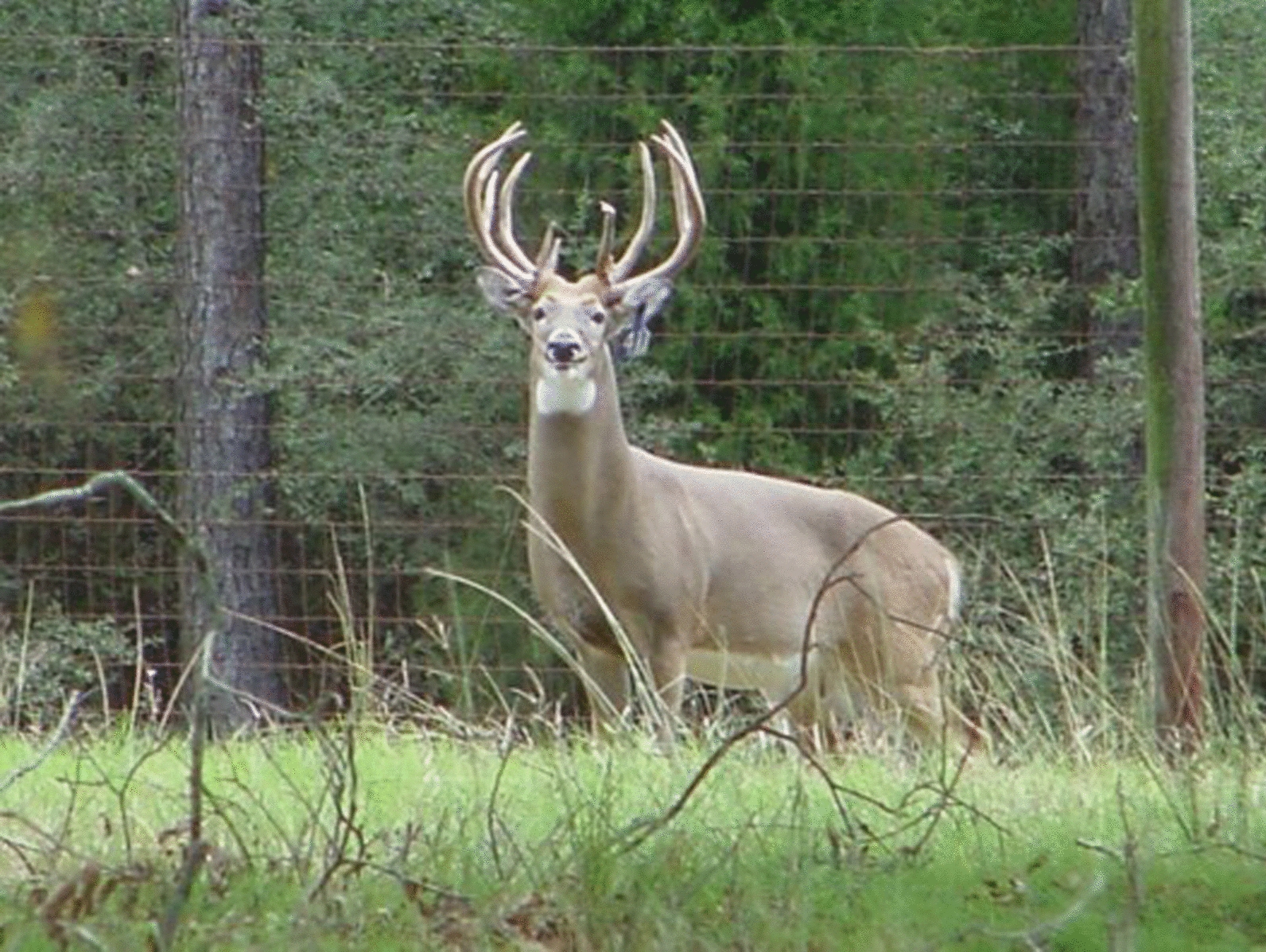

## Background

Bluetongue virus (BTV) and epizootic hemorrhagic disease virus (EHDV) are in the genus *Orbivirus*, family Reoviridae, and are distributed worldwide. These two viruses are transmitted by biting midges of the genus *Culicoides*. Cattle are considered to be primary reservoirs of BTV and EHDV because infections are normally subclinical, with long-lasting viremia. Hemorrhagic disease (HD) in wild ruminants is caused by the infection of BTV or EHDV, with observed mortality rates up to 90% in white-tailed deer (*Odocoileus virginianus*) [1].

The only two previously confirmed vectors of BTV/EHDV in the United States are *Culicoides sonorensis* Wirth and Jones and *C. insignis* Lutz [2, 3]. The species *C. sonorensis* is one of a complex of three species along with *C. occidentalis* and *C. variipennis.* The presence of these three species in the *C. variipennis* complex was confirmed [4]; therefore, in any literature prior to 2000, *C. sonorensis* was synonymous with *C. variipennis*. There have been numerous field studies with reported transmission of BTV and EHDV when *C. sonorensis* was rare or absent in collections [5–8]. Therefore, it is important to determine the local species of *Culicoides* in different areas with BTV/EHDV transmission.

Many studies have been conducted to determine the most efficient light trap type for capturing specimens of *Culicoides*, and black light has most often been found to be superior to incandescent light [9–11]. Although light traps are the gold standard for catching specimens of *Culicoides*, there are potential inadequacies in using these traps as the sole technique in studies on virus transmission. Light trap catches will inherently differ on individual trap-nights depending on light intensity and abiotic factors such as weather conditions or moon phase, and do not necessarily capture all of the species present [12, 13]. For example, Barnard and Jones [14] reported that the diel activity of *C. variipennis* was greatest near sunset but increased near sunrise on some days; however, they noted differences in flight activity periods depending on time of year. In a study by Viennet et al. [15], two species of *Culicoides* were captured in animal-baited traps that were not captured in light traps, and Carpenter et al. [16] found that populations of *C. chiopterus* were substantially underestimated using light traps versus drop traps using live sheep. In addition, Gerry et al. [17] captured 95% of total specimens of *C. obsoletus* in their study from direct aspiration off sheep, which is in contrast to Carpenter et al. [16], where this species was more common in ultraviolet (UV)-baited light traps than on nearby sheep. Therefore, it is important to conduct animal-based collections in areas with orbivirus transmission, especially to determine particular vector species. Although it is often difficult for ethical or logistical reasons, using animal-baited traps in addition to light traps should be considered when conducting surveillance for *Culicoides* midges to validate the species composition and to exclude species not relevant to pathogen transmission.

Studies have shown that the sex, parity, and gravid status of *C. sonorensis* vary in response to trap design [18, 19]. Additionally, infection status potentially influences whether midges are caught in certain trap types. Mayo et al. [20] collected midges using CDC traps with and without black light and directly from cattle and found higher infection rates in *C. sonorensis* midges collected without light and directly from cattle. Subsequently, McDermott et al. [21] used suction traps with and without light and captured significantly more BTV-infected *C. sonorensis* in traps with no light, attributing the results to BTV infections in the eyes of the midges potentially affecting their vision. However, similar studies have not been conducted for other potential vectors of orbiviruses.

This study was conducted to compare trapping methods for collecting potential vectors of orbiviruses in an area of known BTV and EHDV transmission where *C. sonorensis* was previously absent in collections timed with an epizootic of BTV and EHDV transmission [22]. In the first objective, the numbers of species, abundance, and virus infection rates of female *Culicoides* collected in dry ice-baited Centers for Disease Control and Prevention (CDC) traps either with or without black light were compared. A second objective was to compare the abundance of potential vector species at different crepuscular and nocturnal periods using CDC traps that were modified to collect insects in 2-h time intervals from 18:00 to 08:00. The third objective was to compare the number and diversity of species of *Culicoides* captured using five different trap methods: animal-baited drop trap, animal-baited baffle trap, direct animal aspiration, CDC trap with black light, and CDC trap with no light.

## Methods

The study was conducted at the Louisiana State University Agricultural Center Bob R. Jones Idlewild Research Station (BJIRS) near Clinton, Louisiana (30.817954 N, 90.97324 W). The station maintains a reproductive herd of approximately 100 captive white-tailed deer as well as reproductive herds of around 50 crossbred beef cattle and 30 red deer (*Cervus elaphus*). Additionally, wild white-tailed deer roam the 830-hectare facility, which comprises bottomland hardwood and pine forest.

For objective one, six miniature CDC black light traps (model 512; John W. Hock Co., Gainesville, FL, USA) baited with 2 kg of dry ice in igloo containers were deployed approximately 1.5 m aboveground at three different locations 45 min before dusk and collected the next morning 60 min after sunrise, for a total of 240 trap-nights in July–October from 2016 to 2018. The three locations were along a fence outside and adjacent to a white-tailed deer pen where approximately 50 deer were present year-round, along a fence of cattle pasture where 35 cows were present, and in a forested area with a mixture of hardwood and pine trees approximately 0.5 km from the nearest cattle pasture or deer pen. Each location had two trap sites greater than 50 m apart receiving a trap either with or without a light, and for each trapping event the treatment was rotated between light and no light. After collection, the insects were held at 4 °C until the specimens were sorted using a dissecting microscope and a chill table (BioQuip^®^, Gardena, CA, USA). Members of the genus *Culicoides* without visible blood meals were sorted by species through examination of wing patterns using the keys reported by Blanton and Wirth [23]; voucher specimens were confirmed by mounting, dissecting, and clearing, followed by examination of the spermathecae and antennal patterns. Specimens of *C. variipennis* were confirmed by slide mounting and examining the maxillary palps as described previously [4].

The *Culicoides* midges (5–50 individuals) were pooled by trap type, species, site, and date, and the methods described by Becker et al. [22] were used to isolate viral RNA and conduct reverse transcriptase quantitative polymerase chain reaction (RT-qPCR) on the midge pools to test for the presence of BTV and EHDV. Samples were considered EHDV-positive when quantification cycle (Cq) values were less than 40 cycles and BTV-positive when Cq values were 36 cycles or less [24].

A Student *t*-test and Tukey–Kramer post hoc test were performed to compare the mean number of specimens captured overall and for individual species for the light versus no light study. The bias-corrected maximum likelihood estimate (MLE) of the infection rate and 95% confidence intervals for pooled samples were calculated using PooledInfRate software [25]. Fisher’s exact test was performed using GraphPad Prism (version 8.4.3 for Windows, GraphPad Software, San Diego, CA, USA, www.graphpad.com) to compare the proportion of midges infected with BTV/EHDV captured in CDC traps with light or without light.

For objective two, timed collections were conducted using a collection bottle rotator (model 1512; John W. Hock Co., Gainesville, FL, USA) with a CDC miniature black light trap baited with 2 kg of dry ice and collection bottles containing 50 mL of 95% ethanol. The trap was set to collect at eight 2-h intervals beginning at 18:00 and ending at 08:00. The trap was deployed for a total of 15 collection nights in late summer over a 3-year period (2013, 2014, and 2016), five times at each of the three locations used in the light comparison study. Specimens of *Culicoides* were sorted into species following the procedures described above.

For objective three, the trap method comparison study was conducted from August through September 2014, during peak transmission of BTV/EHDV, in an area near a forest edge in the proximity of deer pens and cattle pastures. There were five trapping sites greater than 50 m apart; trap placement was selected at random for the first trial and then rotated in a Latin square design until two full rotations were completed. There were three trap methods that used stanchioned beef calves that were approximately 6 months old and weighed approximately 100 kg, which were randomly assigned to sites for each replicate. The first trap type was an animal-baited baffle trap which was constructed with a wooden frame (1.83 × 1.83 × 1.83 m) covered by 52 × 52 Saran mesh cloth [26]. Three baffles were constructed on the sides to allow insects to enter and feed on a live calf which was stanchioned inside the trap from 45 min before official sunset until 30 min after dark, when the calf was removed from the trap (approximately 1.5 h). Specimens of *Culicoides* were collected using a mouth aspirator with HEPA filter (model 612; John W. Hock Co., Gainesville, FL, USA) the following morning, transported to the lab, and stored at 4 °C. The second trap type was a modified version of the animal-baited drop trap described by Carpenter et al. [16]. A metal frame approximately 2 × 5 × 5 m was constructed to fit around the stanchion and hold up the fine mesh netting, which was dropped around a calf that was stanchioned 45 min before dusk. The net was dropped 10 min before dusk and the animal was removed from the trap 10 min later. Insects were aspirated the next morning from inside the net and stored at 4 °C. The third trapping method was direct aspiration of specimens of *Culicoides* off of a stanchioned calf beginning 45 min prior to sunset using a battery-powered aspirator (model 2888A; BioQuip Products, Rancho Dominguez, CA, USA). Collections were made three times for 10 min each by continuously and thoroughly sweeping the aspirator along the dorsal and ventral side of the calf, neck and head area, and each leg. The collecting cups were transported to the lab and stored at 4 °C. The other two trap types were CDC miniature black light traps (with or without light) baited with 2 kg of dry ice and deployed approximately 45 min before dusk and collected the next morning within 60 min after dawn.

All collected *Culicoides* midges were sorted into species and enumerated. The mean number of all specimens combined, hourly means, and individual means for three species (*C. pusillus*, *C. debilipalpis*, and *C. stellifer*) per trap-night for each of the five trap types was compared using one-way analysis of variance (ANOVA) and Tukey’s post hoc test for separation of means [27].

## Results

In trials for objective one, specimens from 10 species of *Culicoides* (*C. arboricola, C. biguttatus, C. crepuscularis, C. debilipalpis, C. haematopotus, C. stellifer, C. neopulicaris, C. variipennis, C. villosipennis*, and *C. venustus*) were captured using the CDC traps with black light. When using the CDC traps without light, midges from eight of the same 10 species excluding *C. villosipennis* and *C. neopulicaris* were captured.

There was a difference in the overall mean number of specimens captured in CDC traps with black light (17.13 ± 4.51) versus CDC traps without light (6.82 ± 1.54) for data combined for all 3 years; *t*-test (*df* = 239, *F* = 6.12, *P* = 0.014, *n* = 120). For all species except *C. debilipalpis*, the number of specimens captured with light was higher than without light. For two species with larger sample sizes, *C. biguttatus* and *C. stellifer*, the mean number of specimens captured in light traps was significantly higher than in traps without light (Table 1). For all other species, there was no significant difference in the mean number of midges captured with light versus without light. For *C. venustus*, although the traps with light caught over 30 times as many specimens as traps without light, there was a large variance and therefore no statistically significant difference (*P* = 0.11).

**Table 1:** The overall mean ± standard error (SE) of specimens per trap-night and mean from 10 species of *Culicoides* collected in two different types of traps baited with dry ice and deployed overnight during 240 trap-nights from 2016 to 2018 at the Bob R. Jones Idlewild Research Station near Clinton, LA

CDC trap overall mean	Mean no. of specimens captured/trap-night ± SE									
	*C. arb*	C. big*	*C. crep*	*C. vill*	*C. deb*	*C. ven*	*C.var*	C. stel*	*C. neop*	*C. haem*
Black light 17.13 ± 4.51	0.38 ± 0.14	1.12 ± 0.31 ^A^	0.50 ± 0.25	0.07 ± 0.03	2.15 ± 0.54	2.70 ± 1.59	0.54 ± 0.15	9.06 ± 2.68 ^A^	0.28 ± 0.10	0.23 ± 0.08
No light 6.82 ± 1.54	0.20 ± 0.076	0.27 ± 0.09 ^B^	0.13 ± 0.05	0.00 ± 0.00	3.68 ± 1.36	0.11 ± 0.05	0.28 ± 0.08	1.90 ± 0.77 ^B^	0.00 ± 0.00	0.12 ± 0.05

*C. arb*: *C. arboricola*, *C. big*: *C. biguttatus*, *C. crep*: *C. crepuscularis*, *C. vill*: *C. villosipennis*, *C. deb*: *C. debilipalpis*, *C. ven*: *C. venustus*, *C. stel*: *C. stellifer*, *C. var*: *C. variipennis*, *C. neop*: *C. neopulicaris*, *C. haem*: *C. haematopotus*

*Values within columns followed by different letters were significantly different (*P* < 0.05) using the Student *t*-test for *C. big* (*df* = 239, *F* = 6.81, *P* = 0.009, *n* = 120) and for *C. stel* (*df* = 239, *F* = 6.54, *P* = 0.01, *n* = 120)

Pools of field-collected specimens from five out of 10 *Culicoides* species (*C. crepuscularis*, *C. debilipalpis*, *C. haematopotus*, *C. stellifer*, and *C. venustus*) were found positive by RT-qPCR for BTV or EHDV. For the collections of CDC traps with black light captures, a total of 1557 specimens from these five species in 235 pools were tested for BTV and EHDV by RT-qPCR (Table 2); there were 11 BTV-positive and 16 EHDV-positive pools, for an overall bias-corrected MLE of infection rate of 6.9 (confidence interval [CI] 3.80–11.6) for BTV and 9.4 (CI 5.6–14.75) for EHDV for all five species combined. For the traps without light, a total of 909 specimens in 151 pools were tested, resulting in 10 BTV-positive pools and two EHDV-positive pools. The MLE of all five species caught in traps without light was 14.2 (CI 7.39–24.9) for BTV and 2.8 (CI 0.5–9.1) for EHDV. The EHDV infection prevalence for midges was higher in 2017 (16%) than in 2016 (3%) and 2018 (1%).

**Table 2:** Number of EHDV- and BTV-positive pools detected by RT-qPCR out of the total number of pools of specimens from five species of *Culicoides* collected in June–November from 2016 to 2018 using CDC traps with or without black light at the Bob R. Jones Idlewild Research Station near Clinton, LA

Total pools (total specimens)				EHDV-positive pools		BTV-positive pools	
Year	Species	Black light	No light	Black light	No light	Black light	No light
2016	*C. crepuscularis*	2 (20)	1 (11)	0	0	0	0
	*C. debilipalpis*	23 (122)	21 (210)	0	0	2	1
	*C. stellifer*	34 (412)	21 (267)	1	0	0	0
	*C. haematopotus*	6 (9)	8 (10)	0	0	1	0
	*C. venustus*	25 (141)	2 (7)	2	0	0	0
2017	*C. crepuscularis*	9 (19)	7 (9)	1	1	0	0
	*C. debilipalpis*	21 (140)	18 (90)	1	0	0	2
	*C. stellifer*	23 (231)	15 (103)	3	1	2	2
	*C. haematopotus*	3 (6)	1 (2)	2	0	0	0
	*C. venustus*	17 (91)	2 (9)	5	0	2	1
2018	*C. crepuscularis*	7 (12)	2 (4)	0	0	1	0
	*C. debilipalpis*	16 (39)	24 (98)	0	0	1	1
	*C. stellifer*	28 (229)	22 (71)	0	0	1	1
	*C. haematopotus*	5 (10)	3 (5)	0	0	1	1
	*C. venustus*	16 (76)	4 (13)	1	0	0	1
Total		235 (1557)	151 (909)	16	2	11	10

A total of 16 positive pools for EHDV were detected across all five species caught in traps with light; specimens from *C. venustus* accounted for eight positive pools (Table 3). In contrast, only one EHDV-positive pool was detected in *C. crepuscularis* and *C. stellifer* specimens, and no EHDV-positive pools were found for specimens of *C*. *debilipalpis*, *C. haematopotus*, or *C. venustus* collected in traps without light (Table 3).

**Table 3:** Maximum likelihood estimate (MLE) for epizootic hemorrhagic disease virus (EHDV) and 95% confidence intervals from five species of *Culicoides* captured in CDC traps with and without light from 2016 to 2018 at the Bob R. Jones Idlewild Research Station near Clinton, LA

Species	EHDV with light		EHDV no light	
	No. + pools (#spm.)^a^	MLE (CI)^b^	No. + pools (#spm)	MLE (CI)
*C. crepuscularis*	1 (51)	21.6 (0.9–77.9)	1 (24)	47.2 (3.9–271.7)
*C. debilipalpis*	1 (301)	3.9 (0.2–19.1)	0 (398)	na
*C. haematopotus*	2 (25)	70.9 (13.3–211.4)	0 (17)	na
*C. stellifer*	4 (872)	3.7 (1.2–8.8)	1 (441)	4.4 (0.2–21.1)
*C. venustus*	8 (308)	26.9 (12.6–50.9)	0 (29)	na
Total	16 (1557)	9.4 (5.6–14.8)	2 (909)	2.8 (0.5–9.1)

^a^The number of RT-qPCR-positive pools for EHDV (no. + pools) and the number of specimens (#spm.) tested

^b^MLE = bias-corrected maximum likelihood estimate of the infection rate and CI = 95% confidence intervals calculated using PooledInfRate software

Of the five species that tested positive for these viruses, 63% of specimens were captured from CDC traps with light. There was no significant difference (*P* = 0.37) in the proportion of BTV-infected flies caught in traps with light (52%) compared to traps without light (48%). However, 89% of positive pools for EHDV came from traps with light, and there was a difference in the proportion of flies infected with EHDV in traps with light compared to traps with no light (*P* = 0.026).

In trials for objective two, a total of 701 specimens from nine species of *Culicoides* were captured using the timed collection rotator trap: *C. arboricola, C. biguttatus, C. crepuscularis, C. debilipalpis, C. haematopotus, C. stellifer, C. variipennis, C. neopulicaris,* and *C. venustus*. Sunrise was on average at 06:19 and sunset at 20:05, and the average minimum and maximum (min–max) temperatures for each time period were 28–32, 26–28, 25–26, 24–25, 24–23, 23–23, and 23–28 °C. Over 40% of total specimens were captured from 06:00 to 08:00, with an average of 18.8 specimens compared to an average of 2.8 specimens collected in the 18:00–20:00 interval (Table 4). The time period when the most species (five of nine) were collected was after sunset from 20:00 to 22:00. Specimens of the other four species (*C. arboricola, C. biguttatus, C. crepuscularis,* and *C. venustus*) were only caught from 00:00 to 60:00.

**Table 4:** The mean and standard error for total number of specimens, *C. debilipalpis,* and *C. stellifer* specimens captured during 2-h time intervals over 15 trap-nights using a rotator trap at the Bob R. Jones Idlewild Research Station near Clinton, LA

Time interval	Total specimens^a^	*C. debilipalpis*^b^	*C. stellifer*^c^
	Mean ± SE^d^	Mean ± SE	Mean ± SE
18:00–20:00	2.80 ± 0.77^A^	2.13 ± 0.81^A^	0.66 ± 0.19^A^
20:00–22:00	3.53 ± 0.89^A^	1.27 ± 0.46^A^	1.83 ± 0.54^A^
22:00–24:00	4.53 ± 2.21^A^	2.27 ± 1.11^A^	2.13 ± 0.91^A^
00:00–02:00	5.93 ± 2.31^AB^	3.53 ± 1.69^A^	1.53 ± 0.82^A^
02:00–04:00	5.06 ± 1.96^AB^	1.53 ± 0.58^A^	0.33 ± 0.19^A^
04:00–06:00	6.06 ± 2.59^AB^	1.80 ± 0.93^A^	0.00 ± 0.00^A^
06:00–08:00	18.83 ± 7.32^B^	21.66 ± 3.77^B^	1.13 ± 0.51^A^

^a^Total specimens from nine *Culicoides* species, *df* = 104, *F* = 2.77, *P* = 0.02

^b^*df* = 104, *F* = 18.94, *P* = 0.0001

^c^*df* = 104, *F* = 2.06, *P* = 0.06

^d^*SE* standard error. After testing by one-way single-factor ANOVA and Tukey’s separation of means, values across columns followed by the same letter were not significantly different (*P* > 0.05)

Over 95% of the captured specimens were either *C. debilipalpis* or *C. stellifer*. Therefore, analyses of the activity periods of these two species were compared and summarized in addition to the overall number of specimens captured (Table 4). The mean number of *C. debilipalpis* specimens captured during the morning hours (06:00–08:00) was higher (*P* = 0.0001) than that during all other time intervals. However, there was no difference (*P* = 0.06) in the mean number of specimens of *C. stellifer* captured over the time intervals.

**Fig. 1:**
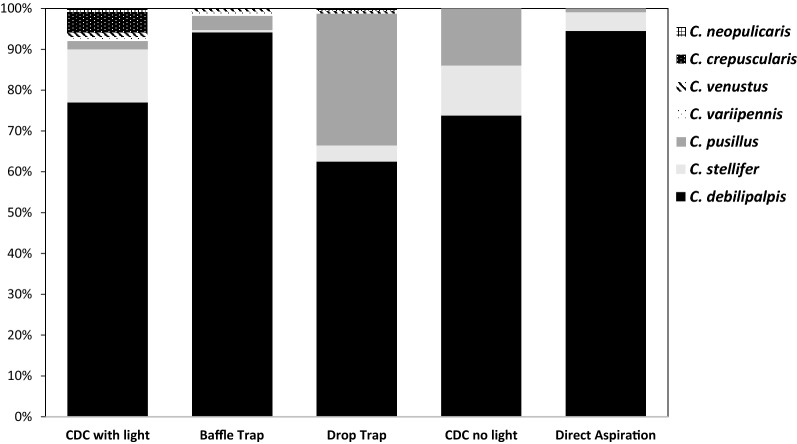
Species percent composition of *Culicoides* midges captured with five different trap methods (CDC with light 7 spp., baffle trap 6 spp., drop trap 5spp., CDC trap without light 3 spp., and direct aspiration from a calf 3 spp.) in 2014 at the Bob R. Jones Idlewild Research Station near Clinton, LA

For the trapping comparison for objective three, a total of 998 specimens from seven species of *Culicoides* were captured during the trapping method comparison study: specimens from four species (*C. arboricola*, *C. venustus, C. variipennis*, and *C. neopulicaris*) accounted for less than 2% of total captured flies and were not included in the analysis. For the other three species (*C. debilipalpis*, *C. pusillus*, and *C. stellifer*) there were no differences (*P* = 0.122) in the mean number of midges captured versus trap type (Table 5). For each trap method, the majority of the captured specimens were *C. debilipalpis*, ranging from 62% of the total capture in the drop trap to 94% of the total for direct aspiration. The second most abundant species in the study was *C. pusillus* (8.8%), followed by *C. stellifer* (4.5%). The abundance of *C. pusillus* was not well represented by black light traps or direct aspiration (Fig. 1, Table 5).

**Table 5:** The mean ± SE (standard error) of specimens overall from seven species combined and for each of the three species of *Culicoides,* number of species, and hourly mean ± SE captured using five different trap types from August through September for a total of 50 trap-nights in 2014 at the Bob R. Jones Idlewild Research Station near Clinton, LA

Trap type	Overall mean ± SE	No. Species	Time	Mean number of specimens captured ± SE		
				*C. pusillus*	*C. debilipalpis*	*C. stellifer*
CDC black light	12.0 ± 5.5	7	19:30–07:00	0.2 ± 0.2	9.6 ± 4.9	1.6 ± 0.9
Baffle trap	61.5 ± 29.8	6	19:30–20:45	2.1 ± 1.2	58.5 ± 29.3	0.3 ± 0.2
Drop trap	19.1 ± 5.8	5	19:30–20:45	6.1 ± 3.8	11.8 ± 5.1	0.7 ± 0.6
CDC no light	16.5 ± 9.6	3	19:30–07:00	3.3 ± 3.2	11.2 ± 6.2	1.8 ± 1.2
Direct aspiration	15.6 ± 5.3	3	19:30–20:15	0.1 ± 0.1	12.6 ± 5.3	1.0 ± 0.4

There was a difference in the number of *Culicoides* species captured using the animal-baited traps (drop trap, baffle trap, and direct animal aspiration) when compared to the CDC traps with or without light (Table 5). Specimens from seven species of *Culicoides (C. debilipalpis, C. neopulicaris, C. pusillus, C. stellifer, C. variipennis* and *C. venustus*, and *C. crepuscularis)* were captured using CDC traps with light (Fig. 1), while all other trapping methods caught a subset of these species. Specimens from six species *(C. debilipalpis, C. neopulicaris, C. pusillus, C. stellifer, C. variipennis*, and *C. venustus*) were captured in the baffle trap*.* Using the drop trap method, specimens from five species (*C. debilipalpis, C. neopulicaris, C. pusillus, C. stellifer*, and *C. venustus*) were captured. Specimens from the same three species (*C. debilipalpis*, *C. stellifer*, and *C. pusillus*) were captured using CDC traps without light and from direct aspiration off the calves.

## Discussion

In the first objective trials, approximately 2.5-fold more midges were captured using the CDC trap with black light compared to without light (*P* = 0.014). The number of specimens captured using light traps varied from year to year, which is typical for multiyear studies on members of the genus *Culicoides* [28]. Over the 3-year trapping period, the range for the yearly mean number of specimens captured in traps without light was 3.4–9.2, and for traps with black light, 14.2–15.2. Potentially, the radius of the trapping range for the traps with light, which was estimated to be 15.25 m for a CDC UV light trap for *Culicoides* midges by Kirkeby et al. [29], could increase the efficacy and reduce variance in trap catch.

Specimens of *C. neopulicaris* and *C. villosipennis* were only captured in CDC traps with lights, but the number of captures was low (Table 1). Significantly more specimens of *C. biguttatus* and *C. stellifer* midges were captured using light. There were nearly fivefold more specimens of *C. stellifer* captured in traps with light, and populations of this species have frequently been found present during epizootics, with specimens being PCR-positive for BTV/EHDV [7, 22]. The only species for which more specimens were captured in traps without light than with light was *C. debilipalpis*. This could serve as important information for future studies, since this species is likely the principal vector for BTV in the southeastern USA. In Louisiana, *C. biguttatus* population peaks normally precede HD outbreaks, and no specimens of this species have been found to be PCR-positive for BTV or EHDV in previous studies [8, 22].

Previous studies suggested that BTV infection status might influence light sensitivity of *C. sonorensis* females and thus impact catches in light traps [21]. In the current study, there were no apparent differences in trap catches of BTV-infected midges between traps with and without light (Table 6). However, since there were 1.7-fold more flies captured using traps with light than without light and the numbers of BTV-positive pools were equivalent, the data do not exclude the possibility that BTV potentially plays a role in light orientation of infected midges of species other than *C. sonorensis*.

**Table 6:** Maximum likelihood estimate (MLE) for bluetongue virus (BTV) and 95% confidence intervals from five species of *Culicoides* captured in CDC traps with and without light from 2016 to 2018 at the Bob R. Jones Idlewild Research Station near Clinton, LA

Species	BTV with light		BTV no light	
	No. + pools (#spm.)^a^	MLE (CI)^b^	No. + pools (#spm)	MLE (CI)
*C. crepuscularis*	1 (51)	16.7 (0.9–79.1)	0 (24)	na
*C. debilipalpis*	3 (301)	12.1 (3.2–32.7)	4 (398)	9.5 (3.1–22.8)
*C. haematopotus*	2 (25)	76.9 (13.8–238.8)	1 (17)	73.4 (4.2–311.5)
*C. stellifer*	3 (872)	2.7 (0.7–7.4)	3 (441)	13.4 (3.6–35.9)
*C. venustus*	2 (308)	9.5 (2.5–25.6)	2 (29)	168.9 (30.4–325.6)
Total	11 (1557)	6.9 (3.8–11.6)	10 (909)	14.2 (7.4–24.9)

^a^The number of RT-qPCR-positive pools for BTV (no. + pools) and the number of specimens (#spm.) tested

^b^MLE = bias-corrected maximum likelihood estimate of the infection rate and CI = 95% confidence intervals calculated using PooledInfRate software

The proportions of infected pools of specimens positive for EHDV and number of specimens tested that were captured in CDC traps without light versus CDC traps with light were different (*P* = 0.026); there were eightfold more EHDV-positive pools from traps with light than from traps without light (Table 3). These novel results provide evidence that EHDV-infected midges are not repelled by light. More research is needed to determine the host-seeking patterns of EHDV-infected midges (including *C. sonorensis*) and to determine whether infection causes light aversion. Given the differences in species diversity and infection rates between traps with and without light that we have shown, the use of traps both with and without light is warranted in studies aimed at vector incrimination for orbiviruses.

The rationale behind the objective two study was that determining peak activity periods of suspected vector species may be important for designing vector control efforts. In this study, there were significantly more specimens of *C. debilipalpis* females collected at sunrise (Table 4) than any other trap period. *Culicoides debilipalpis* has previously been found to be a probable vector of BTV and EHDV in Louisiana and other areas in the southern USA [5, 22]. These data are particularly relevant for vector control in attempts to decrease orbivirus transmission in captive deer herds or for other purposes. The value of the white-tailed deer farming industry was estimated at US$ 3 billion in 2007 [30], and ultralow-volume (ULV) and thermal fog applications of insecticides at white-tailed deer farms to control adult midges are common practices in the USA [31]. The species abundance data from this study indicate that the optimal time for application of insecticide to control *C. debilipalpis* and *C. stellifer*, both of which are probable vectors for BTV/EHDV, would be at sunrise on summer days. This result aligns well with the findings of Fisher et al. [32], which showed that spraying insecticides at lower temperatures in the morning may provide the most effective control.

In objective three trials, there was no significant difference in the mean number of *Culicoides* specimens captured among the five trap methods, but there was a significant difference between the overall hourly capture rates between the baffle trap and the drop trap. The baffle trap collected the most specimens but not the most species (*C. crepuscularis* was absent). Although there was a difference in the number of *Culicoides* species captured for each trap type, there were no *Culicoides* species captured using animal-baited methods that were also not caught in the light traps. In contrast, Viennet et al. [15] showed that two species of *Culicoides* (*C. subfasciipennis* and *C. picturatus*) were captured in animal-baited traps that were not captured in light traps in a study in western France; however, they also reported capturing six species of *Culicoides* in light traps that were not captured in the animal trap. Animal-baited traps are normally used within a narrow time frame, and most previous studies have utilized animal traps in the evening. However, an early morning animal trap method might be appropriate under certain conditions considering the differences in activity periods of different *Culicoides* species we have shown in this study.

Several studies have emphasized the importance of utilizing different trap types in areas of orbivirus transmission, especially when conducting vector surveillance based on known differences in species diversity captured using different trap techniques [17, 20, 21]. Under the conditions of this study, which was conducted during peak orbivirus transmission season [22], we showed that the use of the animal-baited traps (drop trap, baffle trap, or direct aspiration from calves) did not result in the capture of different *Culicoides* species compared with the CDC traps with lights. This finding shows that trapping studies investigating the abundance of different species utilizing a combination of traps with and without lights are valid at the study site and likely other similar habitats.

Although animal-based trap methods may identify species that are not captured in light traps, unintended biases may be introduced into the study when using live hosts to capture insects. Changes in insect behavior in response to a restrained animal, the design and presentation of the trap and trapping protocol, or the influence of human collectors and their associated cues should be carefully considered prior to trapping [33]. Another study [34] found that after testing different animal-baited traps, height above ground level and size of the host animal were more important than the type of host.

In our study, techniques utilized in animal-baited traps that included human interaction may have influenced the outcome, which might be expected. One study [35] showed that specimens of *C. impunctatus* were attracted to certain human odors but repelled by others; furthermore, lights used by human collectors may also alter results. For the drop trap, there were no humans near the trap for the period of time during which midges were allowed to fly in, land, and feed on the calf with no barriers. Of interest, the numbers of specimens of *C. pusillus* collected using the drop trap and CDC trap without light were higher than for other methods (Fig. 1). The efficacy of drop traps is limited by the feeding time of individual flies, which can vary for different species [36], but the advantages of the lack of human-introduced bias could be an important parameter to consider when using animal-based trap methods. The baffle trap has several barriers or obstructions for insect entry into the trap; examples of these challenges are the height of the baffles and lack of a clearly visible host. Once specimens are in the baffle trap, the need to address the feeding time of different species is negated. However, certain midge species, such as *C. stellifer,* are considered to be exophilic, meaning they are reluctant to enter any enclosure or cage to attack a host [23], and our study confirmed this observation (Fig. 1). The number of specimens of *C. debilipalpis*, which is a probable primary vector of BTV in Louisiana, captured using all five trap types was dominant. Six of the seven species that were captured in the CDC traps with light were captured in the baffle trap, while the CDC trap without light caught specimens of only three species during the trials for objective three. Overall, our results indicate that the use of CDC black light traps have value in estimating species diversity under the conditions of the study.

## Conclusions

The results of this study suggest that there is value in selecting proper trapping techniques to detect diversity and infection rates of *Culicoides* species in given areas as well as to determine activity periods for the species present. Our data support other studies which indicated that when searching for *Culicoides* midge vectors of orbiviruses, it is advisable to use traps both with and without light. Once it is determined which species are captured with light traps, the use of a timed collection device can aid in determining the optimal times for control efforts, which we found to be in the early hours of the morning. The CDC light traps caught the most species of *Culicoides*, and the animal-baited traps did not capture any species that were not also caught by light traps. Our results indicate that using traps both with and without light is sufficient for orbivirus vector studies while confirming that all species are represented by light trap catches and no different species are captured using animal-baited traps.

## Data Availability

The datasets used and/or analyzed during the current study are available from the corresponding author on reasonable request.

## Abbreviations

BTV: Bluetongue virus
EHDV: Epizootic hemorrhagic disease virus
CDC: Centers for Disease Control and Prevention
HD: Hemorrhagic disease
UV: Ultraviolet
BJIRS: Bob R. Jones Idlewild Research Station
RNA: Ribonucleic acid
Cq: Quantification cycle
RT-qPCR: Reverse transcriptase quantitative polymerase chain reaction
MLE: Maximum likelihood estimate
HEPA: High-efficiency particulate air
ANOVA: Analysis of variance
CI: Confidence intervals
SE: Standard error
